# Quantifying Species' Range Shifts in Relation to Climate Change: A Case Study of *Abies* spp. in China

**DOI:** 10.1371/journal.pone.0023115

**Published:** 2011-08-24

**Authors:** Xiaojun Kou, Qin Li, Shirong Liu

**Affiliations:** 1 State Key Laboratory of Earth Surface Processes and Resource Ecology, Beijing Normal University, Beijing, People's Republic of China; 2 College of Life Sciences, Beijing Normal University, Beijing, People's Republic of China; 3 Institute of Forest Environment and Ecology, Chinese Academy of Forestry, Beijing, People's Republic of China; University of Swansea, United Kingdom

## Abstract

Predicting species range shifts in response to climatic change is a central aspect of global change studies. An ever growing number of species have been modeled using a variety of species distribution models (SDMs). However, quantitative studies of the characteristics of range shifts are rare, predictions of range changes are hard to interpret, analyze and summarize, and comparisons between the various models are difficult to make when the number of species modeled is large. Maxent was used to model the distribution of 12 *Abies* spp. in China under current and possible future climate conditions. Two fuzzy set defined indices, range increment index (I) and range overlapping index (O), were used to quantify range shifts of the chosen species. Correlation analyses were used to test the relationships between these indices and species distribution characteristics. Our results show that *Abies* spp. range increments (I) were highly correlated with longitude, latitude, and mean roughness of their current distributions. Species overlapping (O) was moderately, or not, correlated with these parameters. Neither range increments nor overlapping showed any correlation with species prevalence. These fuzzy sets defined indices provide ideal measures of species range shifts because they are stable and threshold-free. They are reliable indices that allow large numbers of species to be described, modeled, and compared on a variety of taxonomic levels.

## Introduction

The increasing availability of species distribution models (SDMs) [1], [2], [3], [4], [5], open-access high resolution climate data sets [3], [6], [7], [8], [9], digital species distribution maps, and digital voucher specimen data sets [10], [11], [12], [13], [14] (BGIF http://www.gbif.org; TROPICOS http://www.tropicos.org), make it possible to model species distribution and to predict shifts in species' ranges in response to possible future changes in climate. As a result, many such studies have been carried out [15], [16], [17], [18], [19], [20] and many more can be expected. Studies of changes in biodiversity (either of whole biota or within a certain taxonomic group) will clearly benefit from these methodological advances [21], [22], [23]. However, interpretation of the large quantities of data and predictions likely to be produced will certainly present a challenge. Standardized methods will be required to quantify and compare predicted changes in species distribution. Conventional mapping and visual inspection methods will be inadequate to cope with the amount of data generated, and quantitative indices will be required.

These quantitative indices will also be needed to provide new methods for evaluating SDM results [24], [25], [26]. It has recently become clear that “standard” SDM validation procedures give inadequate or even misleading results [25], [27]. The Kappa index, the area under the curve (AUC) of a receiver operating characteristic (ROC), and other methods involving the partitioning of data sets to build models and validate subsets, are all by nature model fitting techniques [1], [28], [29], [30], [31]. These indices and procedures are valid for evaluating species' range shifts in response to climate change only when model predictions are consistently related to model fitting. Unfortunately, this assumption is rarely met [32]. The performance of a model in predicting outcomes can only be evaluated by examining its predictions. Because the “actual” distribution data in the future are of course lacking, no model can ever be truly validated. However, model uncertainty, model transferability, and ‘hindcasting’ analyses can be performed to evaluate predictions indirectly [24], [26], [30], [33], [34]. Quantitative indices are required to compare models and their predictions statistically.

It is possible to apply traditionally defined indices (discrete map based) to measure range shifts, but difficulties often arise from the fact that most SDMs do not predict conventional maps showing discrete distributions, but instead usually predict either distributions based on assumptions of continuously suitable habitats, or distributions showing the probability of species occurrence [1], [5]. Datum type conversions from continuous to discrete maps are usually performed by classifying predictions into absent/present using a threshold value [32]. However, the choice of threshold values can seriously affect the resulting maps and makes it impossible to compare results between different studies.

Fuzzy set theory provides a promising means of solving this problem because it does not require the use of thresholds [35], [36] but it is a difficult theory to understand and involves complex calculations. The first problem can be addressed through analogies with traditional mapping methods, and the second using GIS programming.

We used two fuzzy set defined indices to quantify range shifts of *Abies* spp. in China to test the hypothesis that shifts in species' ranges caused by climate change vary according to each specie's current distribution [37].

## Methods

### The *Abies* spp. in China

**Figure 1:**
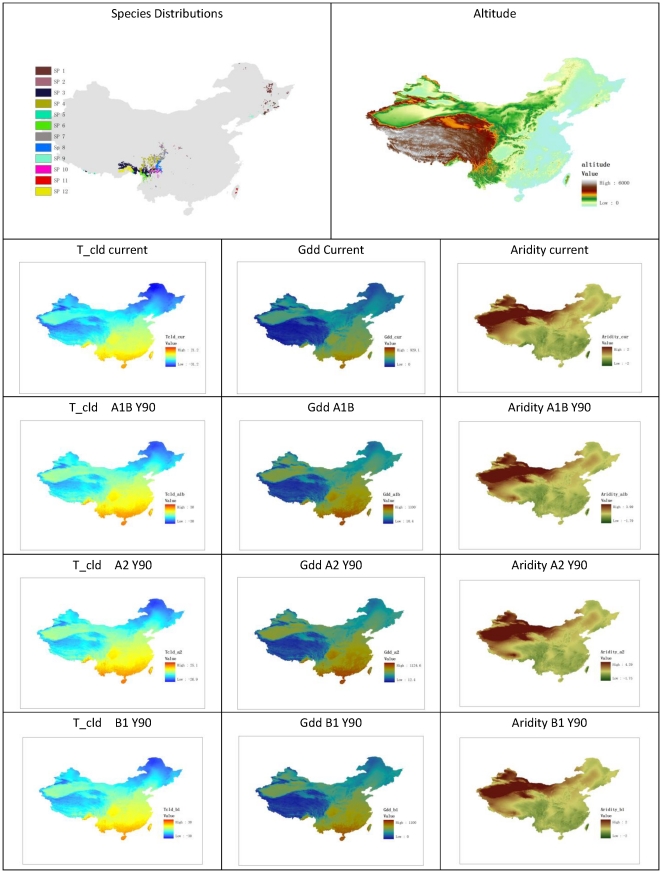
Current Abies species distributions and main environmental factors.

Twenty-eight species (including varieties) of the genus *Abies* (family Pinaceae) occur in China [38], [39], with 12 of them being sufficiently widely distributed to be modeled by SDMs. *Abies* spp. are cold tolerant, moisture-loving, coniferous trees, generally distributed in mountainous areas. In China, *Abies* spp. are concentrated in mountainous areas in the northeast and southwest (Figure 1), where most of the forests occur. They do not occur in northwest China because of their intolerance of aridity (unlike *Picea* spp.). It is not clear whether *Abies* are absent from the central and southeast Chinese plains due to human disturbance or the high ambient temperatures. The relatively large number of *Abies* species, the ease with which they may be located, and the variability in their spatial distribution (both large and small, and compact and dispersed populations occur over a large geographical area) make them a good case study (Table 1).

**Table 1:** General distributional information of the 12 *Abies* Species in China.

Species NO.	SP. 1	SP. 2	SP. 3	SP. 4	SP. 5	SP. 6
Scientific Name	*A.nephrolepis*	*A.fargesii*	*A. georgei*	*A.squamata*	*A.spectabilis*	*A. delavayi*
**No. Cells Occupied**	462	108	1067	598	45	302
**Latitude (Centroid)**	45.678	33.050	29.034	30.39	28.731	27.491
**Longitude (Centroid)**	128.962	106.070	97.240	100.33	90.624	99.041
**Mean Roughness**	0.947	2.728	3.888	3.215	3.947	3.617
**Mean T_cld**	−21.21	−3.60	−3.93	−6.19	−3.95	1.84
**Mean Gdd**	2367.7	2900.5	1954.0	1601.0	1924.9	3310.5
**Mean Aridity**	−0.376	−0.514	−0.452	−0.590	−0.445	−0.610

Species NO.	SP. 7	SP. 8	SP. 9	SP. 10	SP. 11	SP. 12
Scientific Name	*A. fargesii var. faxoniana*	*A. fabri*	*A.holophylla*	*A. forrestii*	*A. awakamii*	*A. delavayi var. motuoensis*
**No. Cells Occupied**	448	300	110	238	39	304
**Latitude (Centroid)**	32.202	29.926	42.127	28.747	24.425	28.866
**Longitude (Centroid)**	102.950	102.550	126.960	101.850	119.950	94.988
**Mean Roughness**	3.488	3.770	1.043	3.563	3.805	4.401
**Mean T_cld**	−5.84	−0.01	−16.57	−1.05	5.38	0.76
**Mean Gdd**	1912.8	3325.9	2665.0	2762.5	3913.8	3268.5
**Mean Aridity**	−0.648	−0.695	−0.508	−0.689	−1.530	−0.573

### Species distribution and environmental data sets

Species distribution data were extracted from the digital Vegetation Atlas of China (1∶1000 000) [40]. The maps have a vector based data format with each vegetation patch represented by a polygon. The smallest polygons that can be detected on the maps are about 1 km^2^.

These maps are compiled from multiple sources, are accepted as the most accurate nationwide Chinese vegetation maps, and show the distribution of vegetation types during the 1970–80s. The vegetation types are classified and named after the first two or three most important community building species typical of each type.

We extracted all the mapped vegetation types containing *Abies* spp. within the vegetation type name (that is where an *Abies* spp. was one of the three main species defining a community). Species with scattered distribution or those that occur only rarely were not included in our species distribution data set. Excluding scattered or rare distributions did not seriously distort our sample ranges because *Abies* spp. are very conspicuous and usually abundant in large patches [39].

The vector maps were firstly converted into raster layers on the ArcInfo workstation (polygrid). The resulting data layers have cells one minute in size. Each species was then aggregated to coarser data layers having a cell size of 5 minutes, to match the resolution of our environmental data.

To project the future shifts in a species' range, we used only climatic data as candidate predictors and ignored other factors such as topography and soil type. The projected ranges therefore reflected only climatic suitability for the species, rather than their true distributions. We chose climatic factors most relevant to plant species distribution, rather than the total climatic data available [41]. The predictive data set used was extracted from BioPlant, a world plant bioclimatic data set with a 10 minute (latitude/longitude) sample resolution [9] and we then downscaled it to 5 minutes using change factor downscaling techniques. The BioPlant data set calculates 15 layers of variables derived from monthly temperature and precipitation data, which were downscaled from GCM model predictions. Change factor downscaling techniques were adopted to obtain a fine scale data set [8], which took full account of the effects of elevation on climatic variables.

In this research, we used the data set derived from the most commonly applied general circulation model (GCM): HADCM3 [3]. Three SRES greenhouse emission scenarios (A1B, A2, B1), and two future time slices, mid-century (20 year means from 2041–2060), and end-century (20 year means from 2081–2100).

Climatic data for calculating bioclimatic variables representing the present were downloaded from the WorldClim data set [7], which has a resolution and interpolating schedule consistent with those used for our future scenarios. The same procedure was applied to calculate the 15 plant biological variables [9].

The standard deviation of detrended elevation data was defined as “topographical roughness” in this study. The 5-minute resolution relative roughness data layer was calculated from SRTM DEM data (http://dds.cr.usgs.gov/srtm/). The SRTM data were first aggregated onto a 1-minute resolution grid, the grid was detrended, and then the focal standard deviations were calculated within each 5×5 focal area cell. The relative roughness was then calculated by dividing the raw roughness by the mean roughness over the whole study area using Arcgis Spatial Analyst.

The whole data set of *Abies* species distributions and environmental variables (current and future scenarios) is available as supporting information (File S1).

### Model implementation

Maxent was chosen to model each species' climate requirements [42], [43], and the fitted models were projected to the future climate scenarios to predict changes in species' ranges. Species sample data were extracted from the grid data derived from the vegetation maps described above. All grid cells that showed the presence of a target species were converted to point data format and 300 cells were selected at random to train the model (this sample limit being selected to avoid over fitting). If a species was present in less than 300 cells, all of the cells were selected. A total of 10 000 background points were selected at random from the entire study area (140 631 cells in total), avoiding cells occupied by the target species.

The mean temperature of the coldest month (T_cld), the growing degree days (GDD) with a threshold of 0°C, and the aridity of the growing season were chosen as environmental variables. Growing season aridity was defined as the ratio of the total potential evapotranspiration during the growing season to the total precipitation in the same period. These three predictors are thought to be the main environmental factors constraining plant species distributions [41]. Grid data of these variables for the present climate and the three climate change scenarios were converted to ASCII data format compatible with Maxent.

All the data layers were mapped in geographical projections (raw latitude and longitude coordinates) and with a resolution of five minutes. The mapping was confined to the Chinese mainland, Hainan, and Taiwan. The other smaller Chinese islands were not included in the analysis.

Model runs were conducted on 12 species, two future time slices (mid-century and end-century), and three climate change scenarios (A1B, A2, B1), using the Maxent batch file running mode. Default settings were adopted and the logistic outputs were recorded.

The AUC, maximum kappa (max_κ), and maximum true skill statistic (max_TSS) indices of all species were calculated to examine the goodness of model fitting.

### Definitions of indices

We adopted the conceptual framework, proposed by Real et al. [36], that uses fuzzy set theory to describe how various conditions favoring a species distribution change in response to changes in climate. Two indices were proposed to measure different aspects favoring a species, namely the increment in favorability (I), and the favorability overlap (O):
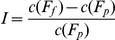


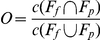
where, *c* represents fuzzy set cardinality, *F_p_* represents the fuzzy set of current distribution, and *F_f_* is the fuzzy set of future distribution.

We extended the meaning of the indices by replacing the membership function from species favorability with potential suitability of species' range. Thus the meanings of the I and O indices are changed to increment and overlap in species' ranges shifts.

The range increment and overlapping indices can be more easily understood in ordinary discrete mapping terms:
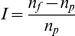


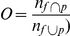
where, *n* represents the number of cells. Subscripts *p* and *f* represent present and future distributions, and subscripts *f∩p* and *fUp* represent combined and overlapping areas of both future and present distributions.

The equivalence of the two definitions can be demonstrated by considering the threshold definition as a special case of a fuzzy set definition with a stepwise membership function. The membership is zero when the occurrence probability is below a given threshold, and one when the probability is above that threshold.

### Calculation of indices and analysis

The I and O indices were calculated for 12 species, at two future time slices (mid-century and end-century), three climate change scenarios (A1B, A2, B1), and using two calculation methods (the threshold method with a threshold of 0.1, and the fuzzy set method. The calculations were all done using an ARCINFO Workstation with arc macro language (AML). GRID algebra was the most commonly applied function in the programming (File S2).

The mean, standard deviation, and maximum and minimum values of the I and O indices of the 12 *Abies* spp. were calculated for each scenario and future time slice. Spearman's rank correlation coefficients were calculated for the species' range shift indices (I and O) compared to the species current distribution parameters (latitude and longitude of the distribution centroid, mean topographical roughness within a species' range, and the number of cells occupied by the species), and the significance of the relationships was tested using the t-distribution. Three significance levels (“very significant” 0.01, “moderately significant” 0.05, and “slightly significant” 0.10) were assigned to the relationships.

## Results

### Performances in model fitting

The area under the curve (AUC) of the receiver operating characteristic, maximum kappa (max_κ), and maximum true skill statistic (max_TSS) are the three most widely used indices to indicate the performance of a model (its discrimination power) for current climate [44]. The AUCs of all the species in the models built using Maxent were greater than 0.980, (average 0.990) showing that the models are powerful discriminators, and that the three selected variables (T_cld, GDD, and growing season aridity) were good predictors of *Abies* spp. distribution. The max_TSS also provided a good model fit, with values for most of the species greater than 0.950. However, max_κ showed model fits that were only moderate, with an average max_κ of around 0.350. TSS and κ were very sensitive to the threshold, with TSS preferring a low threshold and κ preferring a high threshold (Table S1).

### Range shifts of *Abies* spp. in China represented by the I and O indices

**Figure 2:**
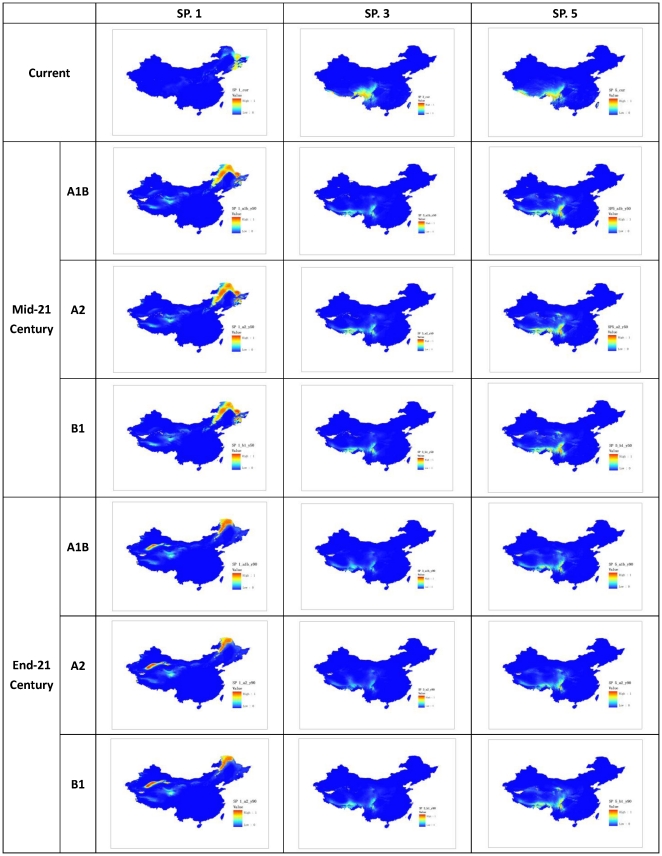
Projected distribution of selected species (Species No. 1, 3, and 5).

**Figure 3:**
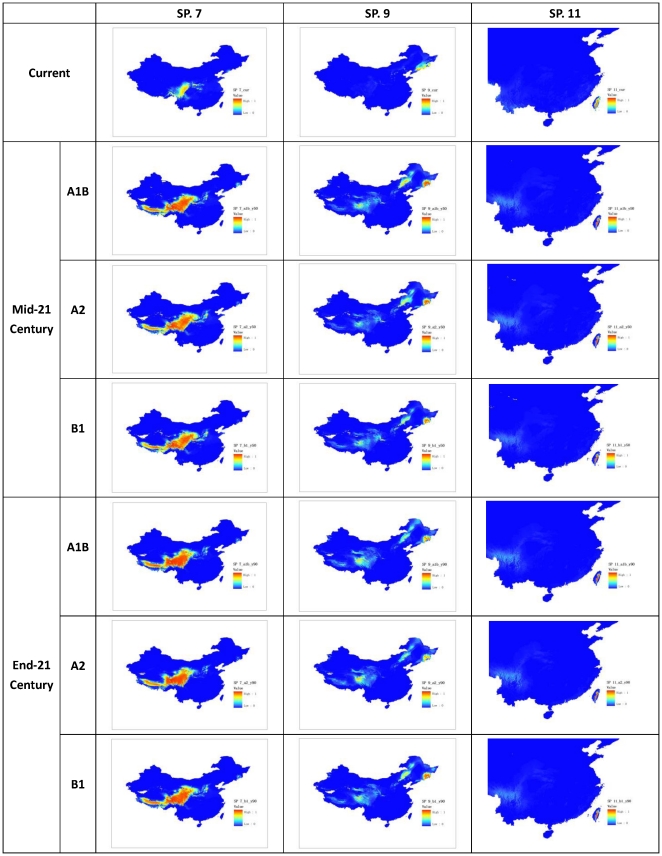
Projected distribution of selected specie (Species No. 7, 9, and 11).

Visual inspection of the modeled maps (Figure 2 and Figure 3) and the plotted I and O index values (Figure S1) show that the index gave meaningful indications of range increments and overlapping that conformed to our intuitive expectations. For example, SP. 1 expanded its range markedly compared with its original ranges and its **I** index values ranged from 1.0 to 2.0 for both future time slices and the scenarios calculated by the threshold and fuzzy set methods. SP. 5 showed most overlap on all occasions (future time slices, climate scenarios, and method of calculation) compared with those of other species, and also had the largest O index values.

The expansion or contraction in range size (**I** index) varied widely among species, with range expansions (**I**>0) more common than contractions (**I**<0). Taking the genus as a whole, the differences among species were much more significant than those within species for the different climate scenarios and future times (Figure S1).

Like the change in range size (**I** index), the range overlapping (**O** index) showed very large interspecies variation, while the variation between scenarios was relatively small. Unlike the change in range size, where the trend between the two future time slices diverged, range overlapping showed very convergent changes, with much smaller overlapping at the end-century than at the mid-century time slice. This means that range overlap decreases with time (Figure S1).

### Correlations between shifts in range and distribution parameters

It is clear from Table 2 that the change in range size (I index) was “very significantly” correlated with latitude, longitude, and mean roughness, and not correlated with the number of cells a species originally occupied. The positive correlations with latitude and longitude mean that *Abies* spp. distributed in northeastern China tended to expand their range much more than their southwestern counterparts. The negative correlation with roughness indicates that *Abies* spp. tended to expand more in flatter areas, or to contract in rougher areas. We were surprised to find that changes in *Abies* spp. range sizes were not correlated with their original areas of distribution. This is unusual because it is generally hypothesized that narrowly distributed species are more vulnerable to climate change. These trends were consistent for all climatic change scenarios, future time slices, and index calculation methods.

**Table 2:** Statistical significances of the correlations between the **I** index and species distribution parameters.

		Mid-century			End-century		
		A1B	A2	B1	A1B	A2	B1
**Threshold Method**	**No. Cell**	O	O	O	O	O	O
	**Latitude**	↑↑↑	↑↑↑	↑↑↑	↑↑↑	↑↑↑	↑↑↑
	**Longitude**	↑↑↑	↑↑↑	↑↑↑	↑↑↑	↑↑↑	↑↑↑
	**Roughness**	↓↓↓	↓↓↓	↓↓↓	↓↓↓	↓↓↓	↓↓↓
**Fuzzy Set Method**	**No. Cell**	O	O	O	O	O	O
	**Latitude**	↑↑↑	↑↑↑	↑↑↑	↑↑↑	↑↑↑	↑↑↑
	**Longitude**	↑↑↑	↑↑↑	↑↑↑	↑↑↑	↑↑↑	↑↑↑
	**Roughness**	↓↓↓	↓↓↓	↓↓↓	↓↓↓	↓↓↓	↓↓↓

Notes: O represents non-significant correlation, ↑↑↑ represents very significant positive correlation, ↓↓↓ represent very significant negative correlation, ↑↑ represents moderately significant positive correlation, ↓↓ represents moderately significant negative correlation, ↑ represents slightly significant positive correlation, and slightly ↓ represents moderately significant negative correlation.

The thresholds for significant categories of “very”, “moderately”, and “slightly” significant are 0.01, 0.05, and 0.10 respectively.

The range overlapping (O index) was clearly not correlated with the number of cells a species originally occupied. It gave “very” to “moderately” significant negative correlations with longitude, while the O index by threshold method gave more “very” significant cases than did the fuzzy set method. The O index gave “moderately” to “slightly” significant positive correlations with roughness, while the O index by the threshold method gave more “moderately” significant cases than did the fuzzy set method. No significant correlation was detected between the O index and latitude in the threshold method cases, but a few cases of slightly significant negative correlations were detected in the fuzzy set cases (Table 3).

**Table 3:** Statistical significances of the correlations between the **O** index and species distribution parameters.

		Mid-century			End-century		
		A1B	A2	B1	A1B	A2	B1
**Threshold Method**	**No. Cell**	O	O	O	O	O	O
	**Latitude**	O	O	O	O	O	O
	**Longitude**	↓↓	↓↓	↓↓↓	↓↓↓	↓↓↓	↓↓
	**Roughness**	↑	↑	O	↑	↑	↑↑
**Fuzzy Set Method**	**No. Cell**	O	O	O	O	O	O
	**Latitude**	↓	O	↓↓	↓	↓	O
	**Longitude**	↓↓	↓↓	↓↓	↓↓	↓	↓↓
	**Roughness**	↑↑	↑↑	↑↑↑	↑↑	↑↑	↑↑

Notes: Same symbols are applied as in Table 2.

By comparing the correlations of the I and O indices to distributional characteristics, the I index shows much more consistency in all climate scenarios, time slices, and index calculation methods.

table-1-captionIn summary, the range size changes and range overlapping were never correlated with the original species distribution areas. The range size changes showed very significant positive correlations with longitude and latitude, and very significant negative correlations with roughness. The range overlapping showed generally negative correlations with longitude, negative or no correlations with latitude, and positive or no correlations with roughness. The original hypothesis is therefore partially upheld with regard to *Abies* spp. in China.

## Discussion

### Why does the threshold problem matter?

As mentioned in the introduction, it is imperative to apply standard indices so that: 1) large numbers of data-rich predicted maps can be adequately summarized; 2) inter-species (or inter-taxa) comparisons can be used to evaluate the overall influence of climate change on biodiversity; and 3) the methodological differences in evaluating model performances, from goodness-of-fit to direct measurement methods, can be examined. Problems in applying the threshold method have presented a major obstacle to the development of such standard indices.

Several methods have been applied to determine appropriate threshold values (arbitrary, maximum corrected rate, maximum kappa, maximum sensitivity plus specialty, or a balance between sensitivity and specialty) [32], [44], [45], and the debate as to which is best continues. It is evident that using different methods to determine thresholds makes comparisons among studies invalid. The stability of these methods is also a major concern. Suppose that the same method is applied to determine a threshold for two different sample subsets of the same species. It is reasonable to suppose that the actual thresholds for each group would be different, so which threshold should be applied? If each model applied used its own threshold, then would the predicted ranges have the same meaning and be comparable? Moreover, if we were to model multi-species range shifts, should we use the same threshold for all species or the “best” threshold for each species? Choosing the best universal threshold for all species would be difficult, and using different thresholds for each would complicate comparisons between the ranges of different species. It is an important property of standard indices that they are threshold-free and this explains the popularity of the AUC index in measuring model performance.

Turnover rate has frequently been used as a quantitative index to measure the effect of climatic change on species' range shifts and changes in biodiversity. This index can be used to measure the overall effects of climate change on a group of species [17], [22], [32], [33], [37]. However, shifts in range at the species level are the basis for all changes at higher taxonomic and community levels. We believe that if predictions are not accurate at the species level, then estimated overall turnover rates must be inaccurate also. The same threshold selection problem that we have discussed above applies in assessing species turnover rate [32]. Arbitrary selection of thresholds, or applying “best” thresholds that are not universally agreed, must add uncertainty to estimates of biodiversity change, and make comparisons among studies problematic. It is possible to formulate turnover rate indices using the same fuzzy set method as for species, and thus make them threshold free.

### Fuzzy set defined indices may be more stable than threshold based ones

Stability is another important property required by a good index. We realized that fuzzy set defined indices are more stable than threshold ones while making visual inspections of index values species by species. Detailed analyses of outliers and inconsistent cases pointed to threshold effects as the primary cause. Close scrutiny of two outliers (SP. 9 and SP. 11) shows that they all contain large areas of low suitability habitat (near threshold 0.1) in the future climate scenarios (Figure 3). The difference between them is that one of those areas (for SP. 9) was slightly greater than the given threshold (0.1) and the other (for SP. 11) was less than the threshold. Even small adjustments of the threshold may result in large differences in predicted ranges. Large areas of near threshold distribution could be the reason for the differences between the two methods of analysis.

It also appears from the overall statistics of the indices (Table S2 and Table S3) that fuzzy set defined indices may be more stable, because the range of fuzzy set defined indices (both I and O) are significantly smaller than the threshold ones, and the standard deviation of the fuzzy set defined O index are smaller than for the threshold defined index.

However, these observations do not conclusively confirm the hypothesis that fuzzy set defined indices are more stable, and further studies are needed. Comparison of the standard deviations of indices in several model runs, at exactly the same model settings, may provide a statistical assessment regarding the absolute stability of these indices.

In summary, the IOMS framework proposed by Real et al. [36] provided a good basis for our study. We calculated two relatively independent indices, I and O, which provide good quantitative descriptions of species' range shift characteristics, and perform well in the example species modeled. By applying these indices to *Abies* spp. in China, we found that: 1) most of the variations in range expansion and distribution overlap in response to climate change are due to interspecies differences rather than the type of climate change scenario modeled; 2) species' range shift characteristics are not correlated with the species prevalence, but show clear correlations with their geographic locations; and 3) species ranges change more (greater range expansion or contraction, and smaller overlaps) in flat areas than in topographically rough areas. Whether these conclusions are unique to *Abies* spp. in China, or represent a general pattern requires further investigation.

## Supporting Information

Figure S1 **I and O indices of Abies species for different scenarios and calculating methods.** In the classification axis, the naming takes the form ##_**_xx. ## represents indices the calculation method of the indices, with Thed representing the discrete method with threshold 0.1, and Fuz the Fuzzy set method. ** represents climate scenarios, taking the value of A1B, A2, or B1. xx represents the future time, with Y50 representing mid-century (2041–2060) and Y90 representing end-century(2081–2100 ). SP. No. follows the definition shown in Table 1.
(DOC)

Table S1 **Model performance indices for 12** ***Abies*** **spp. in China.** Area under the curve of receiver operation characteristic (AUC) , maximum kappa (max_κ), and maximum true skill statistic (max_TSS) are three most widely used indices to indicate model performances (discrimination power) for current climate. A step length of 0.05 on threshold was adopted to determine the thresholds for Max_kappa and Max_TSS.
(DOC)

Table S2 **Statistics of the I index for 12** ***Abies*** **species for three climate scenarios and two future time slices.**
(DOC)

Table S3 **Statistics of the O index for 12** ***Abies*** **species for three climate scenarios and two future time slices.**
(DOC)

File S1 **A rar file of** ***Abies*** **spp. distributions and environmental data layers.**
(RAR)

File S2 **AML codes for calculating the I and O indices.**
(AML)
