# Supplementary material for: Quantifying Species' Range Shifts in Relation to Climate Change: A Case Study of Abies spp. in China
Source: PLoS One. 2011 Aug 24;6(8):e23115. doi: 10.1371/journal.pone.0023115 (PMC3160841; doi:10.1371/journal.pone.0023115)
Supplement: Figure S1 — I and O indices of Abies species for different scenarios and calculating methods. In the classification axis, the naming takes the form ##_**_xx. ## represents indices the calculation method of the indices, with Thed representing the discrete method with threshold 0.1, and Fuz the Fuzzy set method. ** represents climate scenarios, taking the value of A1B, A2, or B1. xx represents the future time, with Y50 representing mid-century (2041–2060) and Y90 representing end-century(2081–2100 ). SP. No. follows the definition shown in Table 1. (DOC) [file pone.0023115.s001.doc]

Figure S1 **I** and **O** indices of *Abies* species for different scenarios and calculating methods

**Figure S1a**

**Figure S1b**
