# Supplementary material for: Quantifying Species' Range Shifts in Relation to Climate Change: A Case Study of Abies spp. in China
Source: PLoS One. 2011 Aug 24;6(8):e23115. doi: 10.1371/journal.pone.0023115 (PMC3160841; doi:10.1371/journal.pone.0023115)
Supplement: Table S1 — Model performance indices for 12 Abies spp. in China. Area under the curve of receiver operation characteristic (AUC) , maximum kappa (max_κ), and maximum true skill statistic (max_TSS) are three most widely used indices to indicate model performances (discrimination power) for current climate. A step length of 0.05 on threshold was adopted to determine the thresholds for Max_kappa and Max_TSS. (DOC) [file pone.0023115.s002.doc]

Table S1. Model performance indices for 12 *Abies* spp. in China

|  | **SP. 1** | **SP. 2** | **SP. 3** | **SP. 4** | **SP. 5** | **SP. 6** |
| --- | --- | --- | --- | --- | --- | --- |
| **AUC** | 0.990 | 0.982 | 0.984 | 0.988 | 0.987 | 0.995 |
| **Max_κ** | 0.360 | 0.204 | 0.459 | 0.350 | 0.250 | 0.399 |
| **Threshold of**  **Max_κ** | 0.60 | 0.80 | 0.60 | 0.55 | 0.90 | 0.60 |
| **Max_TSS** | 0.931 | 0.868 | 0.948 | 0.956 | 0.858 | 0.964 |
| **Threshold of**  **Max TSS** | 0.20 | 0.05 | 0.15 | 0.40 | 0.05 | 0.10 |

|  | **SP. 7** | **SP. 8** | **SP. 9** | **SP. 10** | **SP. 11** | **SP. 12** |
| --- | --- | --- | --- | --- | --- | --- |
| **AUC** | 0.987 | 0.987 | 0.996 | 0.999 | 0.990 | 0.983 |
| **Max_κ** | 0.383 | 0.358 | 0.297 | 0.361 | 0.577 | 0.421 |
| **Threshold of**  **Max_κ** | 0.55 | 0.65 | 0.65 | 0.75 | 0.85 | 0.60 |
| **Max_TSS** | 0.943 | 0.939 | 0.935 | 0.968 | 0.948 | 0.959 |
| **Threshold of**  **Max TSS** | 0.20 | 0.15 | 0.10 | 0.25 | 0.50 | 0.15 |
