# Supplementary material for: Quantifying Species' Range Shifts in Relation to Climate Change: A Case Study of Abies spp. in China
Source: PLoS One. 2011 Aug 24;6(8):e23115. doi: 10.1371/journal.pone.0023115 (PMC3160841; doi:10.1371/journal.pone.0023115)
Supplement: Table S2 — Statistics of the I index for 12 Abies species for three climate scenarios and two future time slices. (DOC) [file pone.0023115.s003.doc]

Table S2. Statistics of the **I** index for 12 *Abies* species for three climate scenarios and two future time slices

|  | | **Mid-century** | | | **End-century** | | |
| --- | --- | --- | --- | --- | --- | --- | --- |
|  |  | **A1B** | **A2** | **B1** | **A1B** | **A2** | **B1** |
| **Threshold**  **Method** | **Mean** | 0.84 | 0.87 | 0.76 | 0.79 | 0.69 | 0.84 |
|  | **Std** | 1.12 | 0.96 | 0.98 | 1.28 | 1.20 | 1.13 |
|  | **Max** | 3.66 | 3.11 | 2.97 | 4.28 | 3.87 | 3.74 |
|  | **Min** | -0.35 | -0.30 | -0.39 | -0.60 | -0.69 | -0.42 |
| **Fuzzy Set**  **Method** | **Mean** | 0.82 | 0.87 | 0.76 | 0.69 | 0.58 | 0.78 |
|  | **Std** | 1.07 | 0.99 | 1.00 | 1.09 | 1.02 | 1.05 |
|  | **Max** | 2.51 | 2.49 | 2.46 | 2.60 | 2.34 | 2.61 |
|  | **Min** | -0.54 | -0.38 | -0.48 | -0.71 | -0.76 | -0.60 |
