# Supplementary material for: Quantifying Species' Range Shifts in Relation to Climate Change: A Case Study of Abies spp. in China
Source: PLoS One. 2011 Aug 24;6(8):e23115. doi: 10.1371/journal.pone.0023115 (PMC3160841; doi:10.1371/journal.pone.0023115)
Supplement: Table S3 — Statistics of the O index for 12 Abies species for three climate scenarios and two future time slices. (DOC) [file pone.0023115.s004.doc]

Table S3. Statistics of the **O** index for 12 *Abies* species for three climate scenarios and two future time slices

|  | | **Mid-century** | | | **End-century** | | |
| --- | --- | --- | --- | --- | --- | --- | --- |
|  |  | **A1B** | **A2** | **B1** | **A1B** | **A2** | **B1** |
| **Threshold**  **Method** | **Mean** | 0.27 | 0.27 | 0.29 | 0.21 | 0.21 | 0.24 |
|  | **Std** | 0.11 | 0.11 | 0.11 | 0.1 | 0.1 | 0.1 |
|  | **Max** | 0.45 | 0.45 | 0.45 | 0.41 | 0.41 | 0.41 |
|  | **Min** | 0.07 | 0.07 | 0.08 | 0.06 | 0.05 | 0.07 |
| **Fuzzy Set**  **Method** | **Mean** | 0.25 | 0.26 | 0.27 | 0.2 | 0.19 | 0.23 |
|  | **Std** | 0.08 | 0.08 | 0.08 | 0.08 | 0.08 | 0.08 |
|  | **Max** | 0.4 | 0.41 | 0.43 | 0.37 | 0.36 | 0.37 |
|  | **Min** | 0.17 | 0.17 | 0.16 | 0.08 | 0.07 | 0.15 |
